# Supplementary material for: Complications and Status Upgrades among Adult Heart Transplant Candidates with Durable LVADs: Waiting 6 to 8 Years for Status Escalation Is Too Long
Source: medRxiv. 2025 Sep 24:2025.09.22.25336215. Preprint. [Version 1] doi: 10.1101/2025.09.22.25336215 (PMC12485994; doi:10.1101/2025.09.22.25336215)
Supplement: Supplement 1 [file media-1.docx]

Supplemental Table 1

| **Characteristic** | **N = 3,881***^1^* |
| --- | --- |
| Durable LVAD Brand |  |
| HeartMate 3 | 3,182 (82.0%) |
| HeartMate II | 92 (2.4%) |
| Heartware HVAD | 570 (14.7%) |
| Evaheart | 6 (0.2%) |
| ReliantHeartAssist 5 | 2 (0.1%) |
| Heartsaver VAD | 4 (0.1%) |
| Worldheart Levacor | 3 (0.1%) |
| Unknown (Categorized as  Durable LVAD) | 22 (0.6%) |
| *^1^* n (%) | |
